# Meteorological Register

**Published:** 1845-07-01

**Authors:** 


**METEOROLOGICAL REGISTER.**—*Station, Buffalo Barracks, N. Y., North Latitude, 42 deg. 53 min.; West Longitude from Washington City, D.C. 1 deg. 53 min. 36 sec.—Altitude of Barometer above Lake Erie 96 feet.*

| 1845 | Barometer. | | | | Detached Thermometer. | | | | | Clearness of Sky | | | | Wind | | | | Clouds. | | | | Wet Bulb | | Rain. | | |
|---|---|---|---|---|---|---|---|---|---|---|---|---|---|---|---|---|---|---|---|---|---|---|---|---|---|---|
| May | Sunrise. | 9 A. M. | 3 P. M. | 9 P. M. | Sunrise. | 9 A. M. | 3 P. M. | 9 P. M. | Daily Mean. | Sunrise. | 9 A. M. | 3 P. M. | 9 P. M. | Sunrise. | 9 A. M. | 3 P. M. | 9 P. M. | Sunrise. | 9 A. M. | 3 P. M. | 9 P. M. | Sunrise. | 3 P. M. | Began. | Ended. | Q'ntity |
| 1 | 29 410 | 29 410 | 29 370 | 29 370 | 53 | 57 | 56 | 48 | 54 5 | 0 | 1 | 8 | 4 | s. 3 | s. 4 | s.w. 5 | s.w. 5 | 0 0 | 0 0 | w 2 | w 2 | 52 | 53 | 1 a. m. | 5 a. m. | 0 06 |
| 2 | 29 410 | 29 46[illegible] | 29 461 | 29 429 | 45 | 50 | 51 | 40 | 48 | 2 | 8 | 10 | 8 | n.w. 5 | w. 4 | s.w. 6 | s.w. 4 | w 3 | w 2 | 0 0 | 0 0 | 42 | 48 | | | |
| 3 | 29 469 | 29 469 | 29 429 | 29 429 | 43 | 52 | 62 | 49 | 52 5 | 5 | 6 | 4 | 1 | s.w. 4 | s.w. 5 | s.w. 5 | s.w. 2 | w 1 | w 1 | w 1 | 0 0 | 44 | 61 | | | |
| 4 | 29 351 | 29 351 | 29 331 | 29 331 | 49 | 53 | 55 | 51 | 52 | 2 | 2 | 2 | 0 | s.w. 3 | s.w. 4 | s.w. 4 | s.w. 3 | w 2 | w 2 | sw 4 | 0 0 | 49 | 56 | 7 30 a. m. | 11 p. m | 0 03 |
| 5 | 29 469 | 29 528 | 29 547 | 29 547 | 37 | 40 | 47 | 41 | 42 | 4 | 5 | 5 | 7 | n.w. 4 | n.w. 4 | s.w. 5 | s.w. 5 | w 2 | w 2 | sw 2 | ne 0 | 36 | 47 | | | |
| 6 | 29 547 | 29 449 | 29 292 | 29 189 | 37 | 48 | 58 | 46 | 47 5 | 9 | 7 | 9 | 8 | s.e. 4 | s.w. 3 | s.w. 4 | s.w. 4 | nw 0 | w 1 | n 0 | 0 0 | 36 | 56 | | | |
| 7 | 29 134 | 29 201 | 29 292 | 29 319 | 47 | 50 | 44 | 39 | 45 5 | 3 | 0 | 1 | 4 | s.w. 3 | n.w. 4 | n.w. 2 | n.w. 5 | sw 2 | w 1 | w 1 | nw 2 | 48 | 42 | 9 a. m. | 3 p. m. | 0 10 |
| 8 | 29 52[illegible] | 29 540 | 29 547 | 29 481 | 29 | 40 | 48 | 44 | 38 5 | 9 | 9 | 7 | 0 | n.w. 3 | n.w. 3 | s.w. 4 | n.w. 4 | nw 0 | nw 0 | nw 0 | 0 0 | 29 | 49 | | | |
| 9 | 29 5[illegible]4 | 29 528 | 29 547 | 29 579 | 45 | 54 | 63 | 48 | 54 | 1 | 8 | 7 | 8 | n.w. 2 | n.e. 3 | n.w. 3 | n.w. 3 | w 2 | se 1 | nw 2 | 0 0 | 44 | 60 | | | |
| 10 | 29 636 | 29 599 | 29 630 | 29 595 | 46 | 62 | 70 | 56 | 58 | 3 | 8 | 7 | 9 | s.e. 2 | s.w. 3 | s.w. 3 | s.w. 2 | w 1 | nw 1 | sw 1 | sw 1 | 45 | 68 | | | |
| 11 | 29 646 | 29 614 | 29 614 | 29 587 | 58 | 64 | 68 | 69 | 63 | 7 | 9 | 7 | 10 | s.w. 3 | s.w. 4 | s.w. 4 | s.w. 3 | sw 1 | 0 0 | w 1 | 0 0 | 57 | 66 | | | |
| 12 | 29 567 | 29 567 | 29 547 | 29 528 | 57 | 70 | 79 | 63 | 68 | 5 | 9 | 6 | 7 | s. 3 | s.w. 3 | s.w. 3 | s. 3 | s 0 | s 0 | sw 1 | n 0 | 58 | 77 | | | |
| 13 | 29 520 | 29 520 | 29 429 | 29 331 | 59 | 75 | 76 | 66 | 67 5 | 3 | 3 | 6 | 3 | s. 1 | s.w. 2 | s.w. 4 | s.w. 3 | s 2 | s 2 | sw 1 | sw 1 | 59 | 73 | | | |
| 14 | 29 292 | 29 256 | 29 213 | 29 134 | 64 | 72 | 74 | 62 | 69 | 7 | 4 | 2 | 1 | s 4 | s. 3 | s.w. 3 | s.w. 3 | sw 1 | sw 1 | sw 1 | w 1 | 63 | 70 | 12 m. | 1 p. m. | 0 03 |
| 15 | 29 134 | 29 232 | 29 205 | 29 213 | 48 | 42 | 46 | 49 | 47 | 1 | 0 | 2 | 6 | n. 5 | n.w. 4 | n.w. 4 | n.w. 4 | n 4 | w 2 | sw 2 | s 3 | 47 | 66 | 4 a. m. | 2 p. m. | 0 30 |
| 16 | 29 449 | 29 481 | 29 496 | 29 508 | 30 | 39 | 54 | 42 | 42 | 8 | 7 | 6 | 0 | n.e. 3 | n. 4 | n. 4 | s.w. 2 | ne 0 | n 2 | n 1 | 0 0 | 30 | 53 | | | |
| 17 | 29 528 | 29 5[illegible]8 | 29 5[illegible]8 | 29 429 | 33 | 52 | 69 | 51 | 51 | 7 | 9 | 4 | 2 | s.e. 3 | s.w. 4 | n. 4 | s.w. 3 | se 0 | se 1 | nw 2 | nw 1 | 33 | 67 | | | |
| 18 | 29 370 | 29 331 | 29 292 | 29 252 | 49 | 61 | 64 | 53 | 56 5 | 2 | 2 | 5 | 7 | s.w. 2 | s.w. 3 | s.w. 3 | s.w. 3 | nw 1 | w 1 | w 1 | nw 1 | 48 | 62 | | | |
| 19 | 29 252 | 29 311 | 29 331 | 29 292 | 51 | 58 | 68 | 61 | 59 5 | 4 | 7 | 1 | 6 | s.w. 2 | s w. 4 | s.w. 3 | e. 4 | nw 2 | nw 1 | nw 1 | w 2 | 50 | 64 | | | 0 01 |
| 20 | 29 331 | 29 331 | 29 410 | 29 410 | 45 | 54 | 59 | 50 | 52 | 6 | 8 | 6 | 4 | n. 2 | n.w. 3 | s.w. 5 | s.w. 3 | n 0 | se 0 | w 1 | w 2 | 45 | 60 | | | |
| 21 | 29 469 | 29 46[illegible] | 29 528 | 29 528 | 39 | 53 | 62 | 59 | 50 5 | 8 | 7 | 8 | 9 | w. 2 | w. 3 | s.w. 4 | s.w. 2 | n 0 | e 0 | w 1 | n 0 | 38 | 62 | | | |
| 22 | 29 5[illegible]8 | 29 449 | 29 394 | 29 402 | 45 | 56 | 48 | 46 | 46 5 | 2 | 0 | 0 | 0 | e. 3 | n.e. 3 | n.e. 3 | n.e. 3 | sw 2 | w 1 | n 2 | n 1 | 44 | 48 | 12 m. | 7 p. m. | 0 30 |
| 23 | 29 449 | 29 449 | 29 429 | 29 415 | 44 | 60 | 62 | 51 | 53 | 1 | 6 | 8 | 6 | n.e. 1 | s.w. 3 | s.w. 4 | s.w. 3 | sw 2 | sw 2 | sw 1 | w 1 | 43 | 60 | | | |
| 24 | 29 469 | 29 477 | 29 469 | 29 465 | 39 | 44 | 57 | 53 | 48 | 7 | 6 | 6 | 2 | n. 3 | n.w. 3 | s.w. 3 | n.w. 4 | se 0 | w 2 | w 1 | w 2 | 39 | 54 | | | |
| 25 | 29 469 | 29 488 | 29 488 | 29 410 | 33 | 45 | 55 | 50 | 44 | 8 | 8 | 7 | 0 | n. w. 4 | n. 5 | s.w. 5 | s·w. 3 | w 0 | se 0 | sw 2 | 0 0 | 32 | 55 | | | |
| 26 | 29 311 | 29 284 | 29 264 | 29 252 | 51 | 54 | 62 | 52 | 56 5 | 1 | 0 | 4 | 7 | s.w. 5 | s.w. 4 | s.w. 4 | s.w. 3 | w 3 | w 2 | w 2 | n 0 | 50 | 60 | 7 30 a. m. | 11 a. m. | 0 05 |
| 27 | 29 351 | 29 370 | 29 331 | 29 272 | 52 | 53 | 68 | 58 | 60 | 3 | 5 | 9 | 9 | s.w. 3 | s.w. 3 | s. 3 | s. 3 | w 2 | w 2 | w 1 | w 1 | 51 | 64 | | | |
| 28 | 29 134 | 29 134 | 29 075 | 29 095 | 64 | 66 | 67 | 58 | 65 5 | 3 | 3 | 2 | 0 | s.w. 4 | s. 5 | s.w. 4 | s.w. 3 | sw 3 | sw 3 | sw 2 | sw 2 | 63 | 64 | 9 a. m. | 9 p. m. | 0 90 |
| 29 | 29 213 | 29 449 | 29 5[illegible]8 | 29 528 | 39 | 40 | 48 | 34 | 43 5 | 0 | 0 | 1 | 10 | n. 3 | n. 3 | n. 2 | n.w. 3 | n 3 | nw 3 | n 3 | 0 0 | 38 | 45 | | | |
| 30 | 29 606 | 29 646 | 29 646 | 29 606 | 32 | 44 | 55 | 42 | 43 5 | 9 | 8 | 9 | 9 | n. 2 | n.e. 3 | n.w. 3 | s.w. 3 | n 0 | w 1 | nw 0 | nw 0 | 34 | 54 | | | |
| 31 | 29 646 | 29 658 | 29 634 | 29 634 | 37 | 53 | 64 | 51 | 59 5 | 6 | 8 | 9 | 10 | s.e. 2 | s.w. 4 | s.w. 3 | s.w. 3 | se 1 | n 1 | n 0 | 0 0 | 37 | 54 | | | |
| M'n | 29 424 | 29 437 | 29 428 | 29 405 | 45 16 | 53 58 | 59 96 | 50 16 | 52 56 | 4 38 | 5 28 | 5 41 | 5 06 | | | | | | | | | 41 64 | 58 00 | | | 1 78 |

REMARKS.—1st—Rainy early A. M. 2d—High winds from 11 A. M. all day. 4th—Showery. 7th—Rainy. 8th—Ice this morning 12th—Halo round the moon at 9 P. M. 13th—Thermometer 83 deg. at 1 P. M. 14th—Rain, accompanied with high winds at noon; silent lightning at 9 P. M. in north 15th—Rain A. M.; thermometer 39 deg. at 11 A. M. 16th—Ice this morning. 17th—Frost. 19th—Shower during the night. 22—Rain P. M. 24—High winds all night. 25—Frost A. M; High winds from 11 A. M. all day. 26—Rain, accompanied with high winds. 28—Rain, accompanied with thunder and lightning all day; rainbow in the east at 6 1-2 P. M. 29—Light snow at 10 A. M. inappreciable. 30—Ice this morning. 31—Frost.

EXPLANATIONS.—The amount of Clear Sky is designated by figures, as follows: 0 signifies no clearness of sky; 1 a very small portion, and so on to 10, which signifies entire absence of clouds or haze. The observations of Wind relate to direction and force; the former is expressed by the customary letters; the latter by figures from 0 to 10, as follows: 0 a calm; 1 barely appreciable breeze; 2 gentle breeze; 3 moderate do.; 4 brisk do.; 5 strong wind; 6 very strong do; 7 storm; 8 great do.; 9 hurricane; 10 violent do. The observations of clouds embrace direction and velocity; the latter is expressed relatively by numbers up to 10, as in the case of wind. The wet-bulb expresses the hygrometric condition of the atmosphere, as determined by evaporation of water upon the bulb of a thermometer covered with fine gauze. The numbers express the lowest point to which the mercury falls, by wetting the bulb and swinging the instrument in the open air. The figures under the head of rain express the vertical depth of water [whether rain or snow] in inches or fractions of inches.

